# Multi-omics driven predictions of response to acute phase combination antidepressant therapy: a machine learning approach with cross-trial replication

**DOI:** 10.1038/s41398-021-01632-z

**Published:** 2021-10-07

**Authors:** Jeremiah B. Joyce, Caroline W. Grant, Duan Liu, Siamak MahmoudianDehkordi, Rima Kaddurah-Daouk, Michelle Skime, Joanna Biernacka, Mark A. Frye, Taryn Mayes, Thomas Carmody, Paul E. Croarkin, Liewei Wang, Richard Weinshilboum, William V. Bobo, Madhukar H. Trivedi, Arjun P. Athreya

**Affiliations:** 1 Department of Psychiatry and Psychology, Mayo Clinic, Rochester, MN USA; 2 Department of Molecular Pharmacology and Experimental Therapeutics, Mayo Clinic, Rochester, MN USA; 3 Department of Psychiatry and Behavioral Sciences, Department of Medicine, Duke Institute for Brain Sciences, Duke University, Durham, NC USA; 4 Department of Quantitative Health Sciences, Mayo Clinic, Rochester, MN USA; 5 Peter O’Donnell Jr. Brain Institute and The Department of Psychiatry at the University of Texas Southwestern Medical Center, Dallas, TX USA; 6 Department of Population and Data Sciences at the University of Texas Southwestern Medical Center in Dallas, Dallas, TX USA; 7 Department of Psychiatry and Psychology, Mayo Clinic, Jacksonville, FL USA

**Keywords:** Depression, Predictive markers

## Abstract

Combination antidepressant pharmacotherapies are frequently used to treat major depressive disorder (MDD). However, there is no evidence that machine learning approaches combining multi-omics measures (e.g., genomics and plasma metabolomics) can achieve clinically meaningful predictions of outcomes to combination pharmacotherapy. This study examined data from 264 MDD outpatients treated with citalopram or escitalopram in the Mayo Clinic Pharmacogenomics Research Network Antidepressant Medication Pharmacogenomic Study (PGRN-AMPS) and 111 MDD outpatients treated with combination pharmacotherapies in the Combined Medications to Enhance Outcomes of Antidepressant Therapy (CO-MED) study to predict response to combination antidepressant therapies. To assess whether metabolomics with functionally validated single-nucleotide polymorphisms (SNPs) improves predictability over metabolomics alone, models were trained/tested with and without SNPs. Models trained with PGRN-AMPS’ and CO-MED’s escitalopram/citalopram patients predicted response in CO-MED’s combination pharmacotherapy patients with accuracies of 76.6% (*p* < 0.01; AUC: 0.85) without and 77.5% (*p* < 0.01; AUC: 0.86) with SNPs. Then, models trained solely with PGRN-AMPS’ escitalopram/citalopram patients predicted response in CO-MED’s combination pharmacotherapy patients with accuracies of 75.3% (*p* < 0.05; AUC: 0.84) without and 77.5% (*p* < 0.01; AUC: 0.86) with SNPs, demonstrating cross-trial replication of predictions. Plasma hydroxylated sphingomyelins were prominent predictors of treatment outcomes. To explore the relationship between SNPs and hydroxylated sphingomyelins, we conducted multi-omics integration network analysis. Sphingomyelins clustered with SNPs and metabolites related to monoamine neurotransmission, suggesting a potential functional relationship. These results suggest that integrating specific metabolites and SNPs achieves accurate predictions of treatment response across classes of antidepressants. Finally, these results motivate functional investigation into how sphingomyelins might influence MDD pathophysiology, antidepressant response, or both.

## Introduction

Major depressive disorder (MDD) is a significant public health challenge and an economic burden [1]. Selective serotonin reuptake inhibitors (SSRIs) are first-line pharmacotherapy for MDD, but more than 50% of patients fail to respond [2]. Multiple studies have investigated combination therapies as a subsequent strategy, with mixed results in terms of improved efficacy [3–6]. Given that the full effects of these medications are often not experienced for months, predicting whether a patient will respond prior to therapy or shortly thereafter would advance clinical practice and future clinical translational research.

Several studies have established the predictability of antidepressant response by employing machine learning strategies [7–15]. Machine learning strategies using clinical and sociodemographic factors predicted response to escitalopram/citalopram with accuracies of 59.6% but could not achieve statistically significant predictions across groups of patients receiving combination antidepressant therapies [12]. Two other independent strategies have demonstrated that augmenting clinical and sociodemographic factors with biological measures can improve the predictability of antidepressant treatment outcomes. First, including plasma p180 metabolomics improved predictability of changes in depression severity in a single cohort of the Combining Medications to Enhance Outcomes of Antidepressant Therapy (CO-MED [5]) subjects receiving mono or combination antidepressant therapies [13]. Second, including six functionally validated genomic biomarkers (i.e., single-nucleotide polymorphisms (SNPs) with mechanisms related to MDD severity, or citalopram or escitalopram response) in the Pharmacogenomic Research Network Antidepressant Medication Pharmacogenomic Study (PGRN-AMPS [16]) improved predictive accuracies of treatment response to >69% in patients treated with either citalopram or escitalopram [7]. These prior studies using either plasma metabolomics or genomics were limited, because they did not demonstrate cross-trial replication of predictions in patients receiving combination pharmacotherapy.

**Fig. 1:**
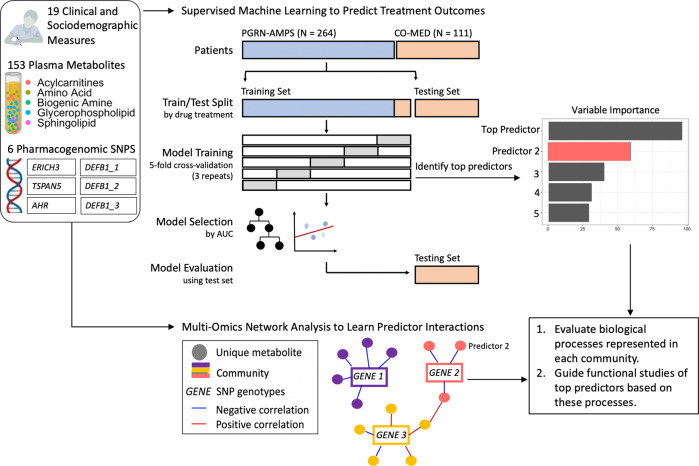
Conceptual overview of model development and evaluation. PGRN-AMPS (Pharmacogenomic Research Network Antidepressant Medication Pharmacogenomic Study) and CO-MED (Combining Medications to Enhance Outcomes of Antidepressant Therapy) participants were partitioned into training/testing groups based upon treatment allocation.

The present study examined data from the PGRN-AMPS and CO-MED studies with a machine learning and multi-omics strategy (Fig. 1) to address key questions. Can machine learning strategies combining plasma metabolomic and genomic measures from MDD patients receiving antidepressant monotherapy (citalopram or escitalopram) achieve statistically significant predictions of response to combination pharmacotherapy? If combining these multi-omics measures improves predictability of response to multiple classes of antidepressants, can multi-omics integration networks elucidate biologically meaningful relationships between metabolomic predictors of antidepressant response and functionally validated genomic biomarkers? This present study hypothesized that augmenting clinical measures (e.g., symptom severity scores) with multiple biological measures (e.g., metabolomics and genomics) might improve the predictability of response to combination antidepressant therapies.

## Materials and methods

### Data sources

MDD outpatients with complete clinical assessments, baseline metabolomics, and genomics from PGRN-AMPS and CO-MED were included in this work (Supplementary Fig. 1 and Supplementary Table 1). Patients were split into training and testing cohorts. The training cohort consisted of patients taking citalopram, escitalopram, or escitalopram plus placebo, whereas the testing cohort comprised patients receiving combination pharmacotherapies (Table 1). Briefly, the PGRN-AMPS 8-week SSRI clinical trial (NCT00613470) enrolled 529 MDD patients who scored ≥14 on the 17-item Hamilton Depression Rating Scale (HAMD-17). Patients received either escitalopram (10 mg/day) or citalopram (20 mg/day). The CO-MED 7-month clinical trial (NCT00590863) enrolled 665 MDD patients who scored ≥16 on the HAMD-17 and who met criteria for either recurrent or chronic (current episode ≥2 years) depression. Patients were randomized to one of the following treatment regimens: (1) escitalopram (up to 20 mg/day) plus placebo, (2) bupropion (up to 400 mg/day) plus escitalopram (up to 20 mg/day), or (3) extended-release venlafaxine (up to 300 mg/day) plus mirtazapine (up to 45 mg/day). These studies were conducted in accordance with the approval of respective Institutional Review Boards to include informed consent. Both studies have been characterized in prior publications [5, 16].

**Table 1:** Clinical and sociodemographic features of training and testing cohorts.

Training and testing cohort				
	*N* = 375 Model Set 1 (Metabolomic)		*N* = 348 Model Set 2 (Multi-omics)	
	Training Set *N* = 298	Testing Set *N* = 77	Training Set *N* = 277	Testing Set *N* = 71
PGRN-AMPS patients (*N*)	264	0	245	0
CO-MED patients (*N*)	34	77	32	71
Sex [% female]	66.1%	71.4%	65.3%	70.4%
Age [mean (SD)]	40.6 (13.3)*	43.3 (11.5)*	41.0 (13.3)	43.4 (11.6)
Years of education [mean (SD)]	14.7 (2.5)*	13.9 (2.4)*	14.8 (2.5)*	13.9 (2.4)*
Race [% White]	93.6%*	77.9%*	96.0%*	77.5%*
Race [% Black or African American]	3.4%*	16.9%*	2.2%*	16.9%*
Race [% Other]	3.0%	5.2%	1.8%	5.6%
Ethnicity [% Hispanic]	2.0%†*	20.8%*	1.4%†*	21.1%*
Depression onset < age 18 years [%]	43.6%	39.0%	41.5%	39.4%
Prior suicide attempts [*N* (%)]	46 (15.4%)	7 (9.1%)	40 (16.3%)	6 (8.4%)
Antidepressants (*N*)	Citalopram (112) Escitalopram (152) Escitalopram + Placebo (34)	Venlafaxine + Mirtazapine (42) Escitalopram + Bupropion (35)	Citalopram (99) Escitalopram (146) Escitalopram + Placebo (32)	Venlafaxine + Mirtazapine (38) Escitalopram + Bupropion (33)
QIDS-C at baseline [mean (SD)]	15.1 (3.4)	15.5 (3.8)	15.1 (3.3)	15.3 (3.7)
QIDS-C response at week 4	47.0%	40.2%	47.7%	42.2%
QIDS-C remission at week 4	26.2%	26.0%	26.7%	28.2%
QIDS-C response at week 8	67.8%	62.3%	69.0%	63.3%
QIDS-C remission at week 8	47.0%	42.9%	48.4%	43.7%

*Significantly different (*p* < 0.05) between training and testing sets according to Mann-Whitney U or chi-square tests. †Ethnicity characterization in PGRN-AMPS is based off data from 205 out of 264 patients, per availability of data.

### Measures and outcomes

Race and ethnicity data were self-reported in both studies (Table 1 and Supplementary Table 1). History of prior suicide attempts was collected as a binary yes/no question; this variable represents the number of unique patients who have had at least one prior suicide attempt (Table 1 and Supplementary Table 1). Depression symptom severity and treatment outcomes were assessed using the Clinician-Rated Quick-Inventory of Depressive Symptomatology (QIDS-C). Response to therapy at 8 weeks was defined as ≥50% reduction in QIDS-C total score from baseline [17]. Remission (reported to characterize the samples in Table 1 and Supplementary Table 1) was defined as achieving a score of ≤5 on the QIDS-C [18].

### Model definitions

We developed two sets of prediction models. The first model set (“Metabolomic Models”) included clinical, sociodemographic, and metabolomic features common to both the PGRN-AMPS and CO-MED studies. All sociodemographic and metabolomic features common to both data sets were included (Table 1 and Supplementary Table 2). Clinical features included baseline and week 4 change of individual QIDS-C items belonging to a “core set” previously defined to extract homogeneous patterns of citalopram/escitalopram response from diverse response trajectories [19] (Supplementary Table 2). Independent studies also demonstrate the predictive utility of QIDS-C baseline totals and early change in total QIDS-C [20]. Therefore, QIDS-C baseline totals and QIDS-C week 4 percent change were also included as clinical predictors (Supplementary Table 2). The second model set (“Multi-Omics Models”) augmented model set 1 with the inclusion of six previously functionally validated SNPs (Supplementary Table 2).

### Metabolomics

Plasma metabolites in both the PGRN-AMPS and the CO-MED cohorts were measured by targeted metabolomics with the AbsoluteIDQ p180 assay platform (BIOCRATES Life Science AG, Innsbruck, Austria) [21] with quality-control and metabolomic profiling as previously published [13, 22, 23]. One hundred and fifty-three metabolites that met quality-control criteria in both the PGRN-AMPS and CO-MED data sets were included. The p180 kit includes all quality control (QC) samples, and calibration and internal standards; therefore, quantifications can be directly compared across studies. This assay detects metabolites from five analyte groups as follows: acylcarnitines, amino acids, biogenic amines, glycerophospholipids, and sphingolipids assayed by use of triple quadrupole tandem mass spectrometry operated in Multiple Reaction Monitoring mode.

### Genomics

Six functionally validated pharmacogenomic SNP biomarkers in or near the *TSPAN5*, *ERICH3*, *DEFB1*, and *AHR* genes, and related to MDD pathophysiology or citalopram/escitalopram response [24–27], were included in the multi-omics models. These six SNPs were selected, because their mechanisms have been extensively characterized with multiple experimental models, including human induced pluripotent stem cells (iPSC)-derived astrocytes and neurons [25, 26]. These SNPs were initially pursued as top signals in metabolomics-informed-genomics studies in PGRN-AMPS depressed patients [24, 27]. The metabolomics-informed-genomics strategy moves beyond heterogeneous clinical disease and outcome phenotypes to help identify genomic and metabolomic variation, which might contribute to individual differences in response to pharmacological agents [28]. In those studies, serotonin and kynurenine concentrations were most significantly associated with SSRI outcomes and baseline depression severity, respectively [24, 27]. Top SNPs that were functionally pursued from the serotonin genome-wide association study (GWAS) were in or near *TSPAN5* (rs10516436) and *ERICH3* (rs696692), and top SNPs from the kynurenine GWAS were in or near *DEFB1* (rs5743467, rs2741130, and rs2702877) and *AHR* (rs17137566). These SNPs were then demonstrated to be predictive of escitalopram/citalopram treatment outcomes in multiple large MDD cohorts [7].

These six SNPs are not exhaustive of the list of SNPs, which may be predictive of antidepressant outcomes. For example, the International Study to Predict Optimized Treatment in Depression found a significant main effect of the rs10245483 SNP, which alters P-glycoprotein expression in lymphoblast cells [29], on predicting remission [30]. To test whether the predictability of outcomes using PGRN-AMPS-derived metabolomic-informed-genomic SNPs may be augmented with rs10245483, we conducted additional experiments incorporating this SNP (Supplementary Table 3).

PGRN-AMPS genotyping was done using Illumina human 610-Quad BeadChips (Illumina, San Diego, CA, USA), with imputation and QC as previously reported [27]. CO-MED genotyping was done using Illumina Quad, Human Omni 2.5 bead chip, as previously published [31]. One of the six SNPs was genotyped in the CO-MED sample with a LooRsq > 99% and the remaining five SNPs were imputed using the Michigan Imputation Server with an imputation *R*^2^ > 97.5% and a call rate > 99%.

### Machine learning strategy

#### Data preprocessing

Features with ≥10% missingness and individuals missing ≥20% of features were excluded (Supplementary Fig. 1). Serotonin and body mass index (BMI) were among the features that were excluded for having ≥10% missingness. This yielded a complete data set, except for one missing taurine value, which was imputed using K-nearest neighbor’s imputation. Metabolites were transformed by the Yeo-Johnson transformation then centered at zero and scaled to unit variance. Nominal variables were converted to binary numerical values. Additive allele effects were assumed for SNPs.

#### Prediction model development

**Fig. 2:**
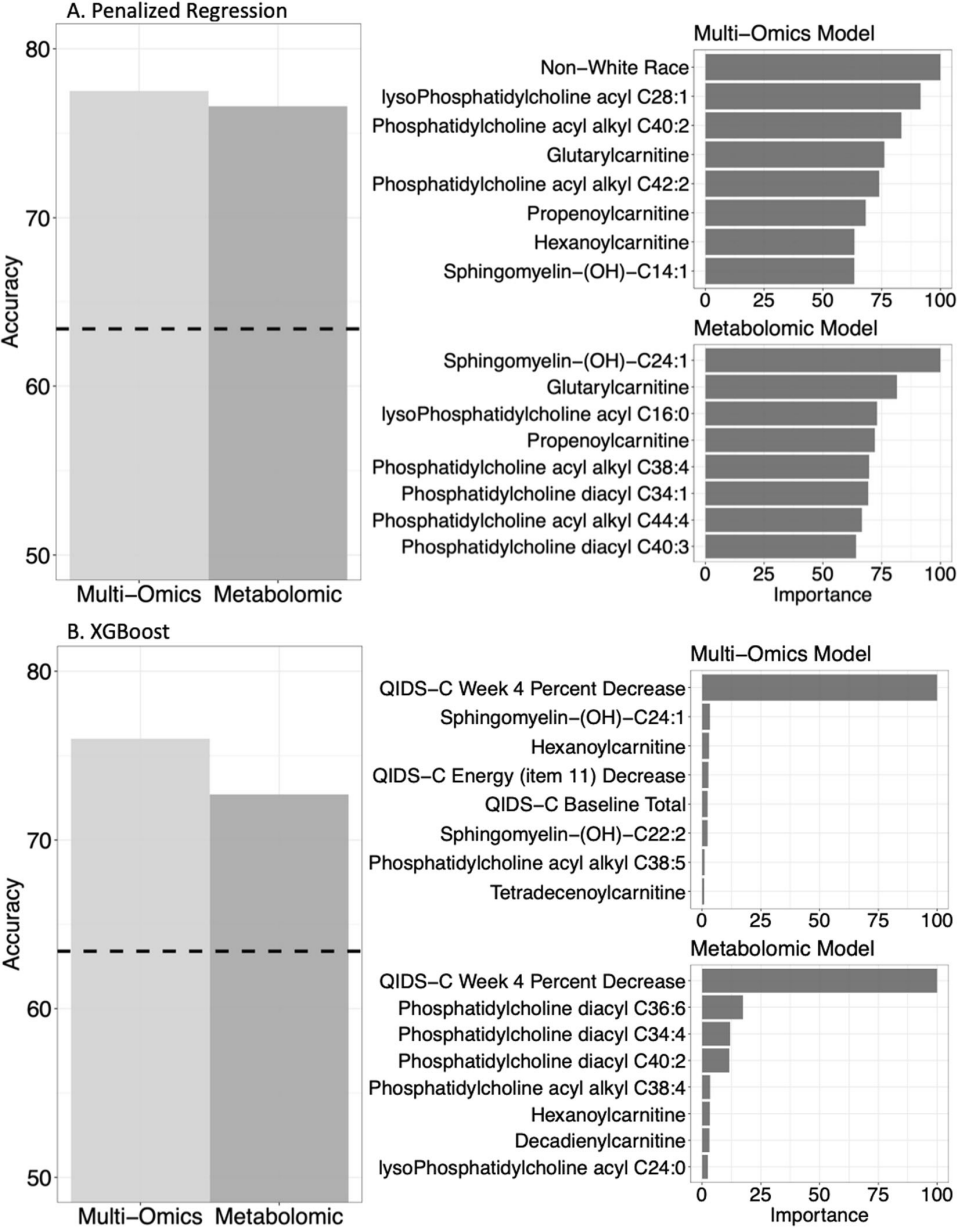
Comparison of test-set accuracies for metabolomic and multi-omics models, with variable importance. Dashed line: null information rate (NIR). The NIR represents the response rate of 63.4% at 8 weeks of therapy. This serves as a benchmark to assess the significance of prediction accuracy.

Models predicting antidepressant response at 8 weeks of therapy were trained on a treatment-homogenous set of patients taking the following SSRI monotherapies: citalopram, escitalopram, or escitalopram plus placebo (Fig. 1 and Table 1). In order to minimize the chance of overfit, estimate prediction performance, and tune model hyperparameters, we used fivefold cross-validation with three repeats. For robustness, we also conducted experiments utilizing threefold and tenfold cross-validation with three repeats (Supplementary Table 4). Both linear and nonlinear algorithms were tested. As a linear penalized regression approach using clinical and metabolomic features successfully yielded predictive insights into change in QIDS Self-Report score in the CO-MED data set [13], we tested penalized regression performance in our combined cohorts. We then tested extreme gradient-boosted decision tree-based ensembles (XGBoost) as nonparametric models. Nonparametric models identify possible nonlinear relationships among predictors (e.g., metabolites and age [32]), while predicting treatment outcomes. These models both have the advantage of being tolerant to multi-collinear data [33]. For all models, upsampling was used to correct for the class imbalance (67% response, 33% non-response). Upsampling injects minority class data points into the training set, equalizing the counts of both classes and preventing model inclination towards the majority class [34]. Variable importance plots were generated to show top predictors of antidepressant response (Fig. 2). We report the expanse of the grid search and tuned hyperparameters for both approaches in Supplementary Table 5.

The best-trained models based on the area under the receiver operating characteristic curve (AUC) during cross-validation were tested on an independent set of cases receiving combination pharmacotherapy (Table 2A). AUC is a widely used classification performance metric that ranges from 0.5 (random guessing) to 1.0 (perfect prediction). Model performance on the test set was evaluated according to accuracy, sensitivity, specificity, and AUC. Preprocessing and machine learning analyses were performed using the tidymodels package version 0.1.2 [35] implemented in R 4.0.3 [36] using RStudio version 1.3 [37].

**Table 2:** Metrics of prediction performance.

Prediction model metrics				
2A: Models trained with PGRN-AMPS escitalopram, PGRN-AMPS citalopram, and CO-MED escitalopram + placebo patients.				
	Model Set 1 (Metabolomic)		Model Set 2 (Multi-omics)	
	XGBoost	Penalized regression	XGBoost	Penalized regression
Testing-set metrics				
AUC	0.76	0.85	0.83	0.86
Accuracy	0.727	0.766	0.761	0.775
NIR	0.62	0.62	0.63	0.63
*p*-Value	0.053	0.0045	0.017	0.0067
Sensitivity	0.75	0.69	0.69	0.71
Specificity	0.69	0.90	0.88	0.88
Training-set metrics (in cross-validation)				
AUC	0.69	0.69	0.68	0.72
Accuracy	0.64	0.66	0.67	0.65
NIR	0.68	0.68	0.69	0.69
**2B: Models trained with PGRN-AMPS escitalopram and PGRN-AMPS citalopram patients**.				
Testing-set metrics				
AUC	0.75	0.84	0.74	0.86
Accuracy	0.753	0.753	0.732	0.775
*p*-Value	0.026	0.026	0.085	0.0067
Sensitivity	0.65	0.73	0.80	0.71
Specificity	0.93	0.79	0.62	0.88
Training-set metrics (in cross-validation)				
AUC	0.68	0.68	0.72	0.72
Accuracy	0.64	0.65	0.67	0.68
NIR	0.69	0.69	0.70	0.70

For the penalized regression models, cartesian grid search was used to tune penalty and mixture hyperparameters, with 20 evenly spaced penalty values ranging from 1*e* − 6 to 10 and mixture values of 0, 0.05, 0.2, 0.4, 0.6, 0.8, and 1. For the XGBoost models, we tuned the number of trees, tree depth, minimum number of data points to split a node, learning rate, loss function reduction, and sample size using Bayesian optimization and a stopping criterion of no improvement over 30 iterations.

#### Cross-trial replication experiment

We conducted an additional analysis to determine whether models trained with citalopram or escitalopram-treated patients (PGRN-AMPS monotherapy-treated patients) could predict outcomes in the CO-MED combination therapy-treated patients (Table 2B). This additional analysis enhances the validity of the cross-trial prediction performance. These additional models were trained and tested according to the previously outlined approaches.

#### Multi-omics integration

The goal of the multi-omics integration analysis was to understand the interaction of predictive plasma metabolomic measures with functionally validated SNPs (Fig. 1). The multi-omics integration network analysis tool xMWAS [38] took all 153 metabolomic, 6 genomic, and clinical response features from all 348 participants as input (Supplementary Table 1). Network analysis was performed using sparse partial least squares (sPLS) regression analysis, a multivariate approach for data integration, with |*r*| > 0.1 and *p* < 0.05. sPLS simultaneously performs variable selection and feature integration, and was originally designed for scenarios with highly correlated variables [39]. As output, the community detection method in xMWAS generates a network of communities (sub-networks) comprising nodes (highly correlated SNPs and metabolites), which are tightly associated within the community but sparsely associated to nodes of other communities [40]. We hypothesized that we would find interactions between kynurenine and the *DEFB1* and *AHR* SNPs, as these SNPs were originally pursued as top signals from a GWAS for kynurenine [24]. Therefore, we performed a second multi-omics integration analysis excluding kynurenine, to assess changes in community membership in the absence of this known strong correlation. Multi-omics integration uncovers associations (via community membership) between top metabolite predictors and functionally characterized SNPs.

## Results

### Prediction of combination therapy-treated patients with citalopram-, escitalopram-, and escitalopram + placebo-treated patients

#### Model training

In the citalopram/escitalopram/escitalopram + placebo monotherapy training set (which used repeated cross-validation to train prediction models), clinical, sociodemographic, and metabolomic features of the “metabolomics models” predicted response to treatment at 8 weeks with an AUC of 0.69 in both machine learning algorithms. When metabolomic and clinical predictor variables were further augmented with six pharmacogenomic SNPs (“multi-omics models”), the training-set prediction AUCs were 0.68 (XGBoost) and 0.72 (penalized regression)—representing an improvement of 0.03 for penalized regression (Table 2A).

#### Model testing

Using the “metabolomics models” feature set, the best-trained classifiers predicted response to combination antidepressant therapies at 8 weeks with accuracies of 76.6% (*p* < 0.005; AUC: 0.85) and 72.7% (*p* = 0.053; AUC: 0.76) for penalized regression and XGBoost, respectively. Using the “multi-omics models” feature set, accuracies improved to 77.5% (*p* < 0.01; AUC: 0.86) and 76.1% (*p* = 0.017; AUC: 0.83) (Table 2A).

### Prediction of combination therapy-treated patients with citalopram and escitalopram-treated patients

#### Model training

In the citalopram/escitalopram monotherapy training set (comprising only PGRN-AMPs patients and which used repeated cross-validation to train prediction models), clinical, sociodemographic, and metabolomic features of the “metabolomics models” predicted response to treatment at 8 weeks with an AUC of 0.68 in both machine learning algorithms. When metabolomic and clinical predictor variables were augmented with six pharmacogenomic SNPs (“multi-omics models”), the training-set prediction AUCs increased to 0.72 for both XGBoost and penalized regression—representing an improvement of 0.04 for both algorithms (Table 2B).

#### Model testing

Using the “metabolomics models” feature set, the best-trained classifiers from PGRN-AMPS subjects receiving citalopram or escitalopram monotherapy predicted response to combination antidepressant therapies at 8 weeks with accuracies of 75.3% (*p* = 0.026; AUC: 0.84) and 75.3% (*p* = 0.026; AUC: 0.75), and for penalized regression and XGBoost, respectively. Using the “multi-omics models” feature set from these subjects, accuracies changed to 77.5% (*p* = 0.0067; AUC: 0.86) and 73.2% (*p* = 0.085; AUC: 0.74) (Table 2B)—representing an improvement of 2.2% for penalized regression.

#### Top predictor variables

The top predictors were calculated using the models trained on escitalopram, citalopram, or escitalopram + placebo-treated patients, to maximize the number of patients in the variable importance calculations. The top predictors varied by algorithm and feature set, but hydroxylated sphingomyelins, glycerophospholipids, clinical/sociodemographic features, and acylcarnitines, and were all represented (Fig. 2). Although SNPs were not among top predictors in both approaches, augmenting the models with SNPs increased the predictive importance of clinical/sociodemographic features (ethnicity, baseline depression, and change in energy). In addition, in the XGBoost models, change in QIDS-C score was a top predictor of treatment outcomes and inclusion of SNPs increased the predictive importance of hydroxylated sphingomyelins.

#### Multi-omics network integration

Integrative network analysis was used to establish relationships between top metabolite predictors (i.e., hydroxylated sphingomyelins) and SNPs whose MDD-related mechanisms have been functionally characterized. The community detection algorithm in this analysis identifies communities as groups of nodes (metabolites and SNPs), which are tightly associated with nodes inside but loosely associated with nodes outside the community [40].

**Fig. 3:**
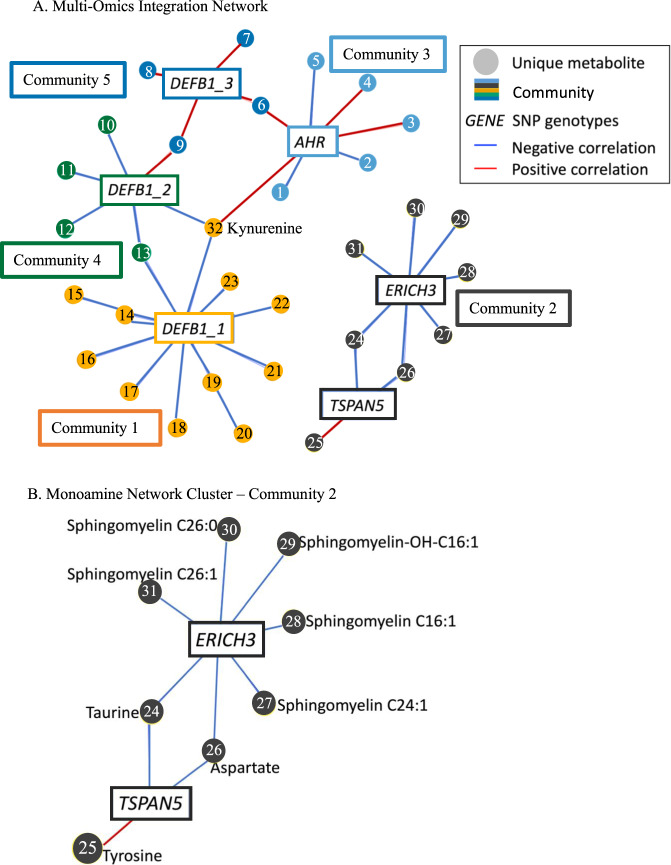
Multi-omics integration network analysis. **A** Multi-omics integration network. Each metabolite is labeled with a number. Metabolite names corresponding to these numbers can be found in Supplementary Table 6. Correlation values between metabolites and SNPs can be found in Supplementary Table 7. **B** Labeled and enlarged community-2 containing sphingomyelins. Represented genotypes are: *TSPAN5* (rs10516436), *ERICH3* (rs696692), *AHR* (rs17137566), *DEFB1_1* (rs5743467), *DEFB1_2* (rs2741130), *DEFB1_3* (rs2702877).

The input to the network analysis included all 153 metabolites, 6 SNPs, and treatment response labels. The analysis identified five communities of statistically related (|*r*| > 0.1 and *p* < 0.05) entities, comprising 32 metabolites and 6 SNPs (Fig. 3). All communities contained both SNPs and metabolites. SNPs in or near the *DEFB1* and *AHR* genes clustered into communities 1, 3, 4, and 5, whereas SNPs in or near *TSPAN5* and *ERICH3* clustered with sphingomyelins and amino acids in community-2 (Fig. 3B). The *ERICH3* SNP in community-2 correlated negatively with various sphingomyelins and a hydroxylated sphingomyelin. Both the *TSPAN5* and *ERICH3* SNPs in community-2 correlated negatively with taurine and aspartate, and the *TSPAN5* SNP correlated positively with tyrosine. Sphingomyelins were absent from communities 1, 3, 4, and 5, where glycerophospholipids, amino acids, and acylcarnitines were represented (Fig. 3 and Supplementary Table 6). Kynurenine clustered with the *DEFB1* and *AHR* SNPs, as expected, as these SNPs were originally pursued from a GWAS for kynurenine (Fig. 3A). When the analysis was repeated without kynurenine, community membership remained consistent (Supplementary Fig. 2). Pearson’s correlation coefficients between SNPs and metabolites can be found in Supplementary Table 7.

## Discussion

The present findings integrated clinical and multiple biological measures to achieve 77.5% accuracy (*p* = 0.0067; AUC = 0.86) in predicting cross-trial outcomes to combination antidepressant therapy. The multi-omics driven augmented prediction performance marks an improvement over prior work in that (1) baseline clinical and sociodemographic measures alone previously achieved 51.4% accuracy in the CO-MED cohort [12] and (2) there is no prior evidence that omics-based models can achieve cross-trial replication in predicting response to combination antidepressant pharmacotherapies. Up to this point, it had been demonstrated that augmenting clinical and sociodemographic measures with either genomics [7] or metabolomics [13] alone improves predictability of treatment outcomes—this work demonstrates for the first time that incorporating SNPs and metabolites together can achieve cross-trial replication in predicting response to combination antidepressant therapies. Cross-trial replication was achieved despite characteristic differences in the PGRN-AMPS training and CO-MED testing cohorts, including race, ethnicity, and antidepressant therapy (Table 1). PGRN-AMPS patients represent a population based in the Upper Midwest of the United States, whereas CO-MED patients represent a more diverse population recruited from 15 sites throughout the United States. These findings encourage future antidepressant studies to assay a wider range of biological measures (e.g., proteomics, transcriptomics, and epigenomics), which might not only continue to improve predictability but might also advance our molecular understanding of MDD pathophysiology, antidepressant response, or both.

The six SNPs included in this work are not meant to be exhaustive or definitive of the entire set of SNPs, which are predictive of antidepressant therapy outcomes. Instead, they were selected, because their MDD-related mechanisms have been characterized extensively [24–27] and they previously increased the predictability of SSRI treatment outcomes compared with models utilizing clinical or sociodemographic factors alone [7]. Incorporating an additional P-glycoprotein SNP (rs10245483) yielded no further improvements in predictability (Supplementary Table 3). This may be related to drug-specific effects of rs10245483 [30], although future experiments would be needed to clarify this. Future experiments should aim to identify additional SNPs, which may synergize with the six metabolomics-informed-genomics SNPs included in this work to improve predictability in the future.

The multi-omics network analysis aimed to elucidate how the combination of these SNPs and metabolites (the “multi-omics models”) continued to improve the predictability of treatment outcomes. The network analysis clustered *TSPAN5* and *ERICH3* in community-2, separate from communities that include *DEFB1* and *AHR* (Fig. 3). Human iPSC-derived astrocytes and neurons, and rodent models have demonstrated that *DEFB1* and *AHR* regulate central and peripheral inflammation via modulation of the kynurenine pathway, whereas *TSPAN5* and *ERICH3* impact monoamine neurotransmission via multiple mechanisms (reviewed in ref. [41]) [24–27]. These monoamine-related SNPs cluster with the monoamine-related metabolites taurine and tyrosine (Fig. 3). Tyrosine is a precursor to catecholamine monoamines, whereas taurine is a natural analog to the alcohol-use-disorder treatment acamprosate, a drug which impacts monoamine-pathway enzyme concentrations [26]. Incorporating both SNPs and metabolites (“multi-omics” models) likely improved predictive accuracy over single-omics models through complimentary biological measurements of processes perturbed in MDD, including monoamine neurotransmission [38] and inflammation [42, 43].

This current analytical workflow ranks biomarkers by their contributions to improved predictions and elucidates opportunities for laboratory-based studies accordingly. Based on these results, laboratory-based experiments might explore individual variation in hydroxylated sphingomyelins, as they are both top predictors of treatment response and are also associated with the monoamine neurotransmission community-2 (Fig. 3B). The importance of hydroxylated sphingomyelins in predicting MDD treatment outcomes is also highlighted by the following evidence: hydroxylated sphingomyelins were represented in a signature of ketamine treatment response [44] and the baseline ratio of hydroxylated to non-hydroxylated sphingomyelins, as well as a larger change in this ratio by 12 weeks of therapy, predicted greater reduction in depressive symptoms with escitalopram or combined medication [13]. Our current analytical approach extends these findings to associate hydroxylated sphingomyelins with markers of monoamine neurotransmission, providing a foundation for follow-up mechanistic studies.

Sphingomyelins are dominant lipids in the outer leaflet of the plasma membrane of most cells and are especially prevalent in the brain [45, 46]. Their hydrogen-bonding capabilities confer unique functional properties distinct from glycerophospholipids and other membrane lipids [45]. Sphingomyelins help maintain the structural integrity of lipid rafts [47], which are highly ordered membrane domains involved in cell signaling [48, 49]. Hydroxylated sphingomyelins are a more polar species that promote fluidization of lipid rafts [47]. Sphingomyelin species, including hydroxylated and long-chain sphingomyelins, affect a multitude of processes such as the regulation of endocytosis [45], ligand binding to the serotonin_1A_ receptor [50, 51], and receptor-mediated ligand uptake [45]. The sphingomyelin-ceramide system has also been demonstrated to play a role in antidepressant response through mechanisms related to autophagy [52].

Given the known roles of sphingomyelins at the plasma membrane and the correlations that we observed between sphingomyelins and the *TSPAN5* and *ERICH3* SNPs in our network analysis, we hypothesize a functional, as well as a statistical relationship between hydroxylated sphingomyelins (or their isomeric/isobaric relatives) and *TSPAN5/ERICH3* mechanisms of pathophysiology in MDD. Both TSPAN5 and ERICH3 physically interact with neurotransmitter vesicle-associated proteins; therefore, previous work suggests that neurotransmitter vesicle biogenesis and/or function is a mechanism by which both genes influence monoamine availability [25, 26]. Vesicles store monoamine neurotransmitters until exocytosis at the plasma membrane [53]. The plasma membrane itself plays a pivotal role in the neurotransmitter life cycle, influencing synaptic vesicle synthesis, storage, release, transport, and degradation [54]. As prevalent polar lipids in the outer leaflet of the plasma membrane [47], sphingomyelins (hydroxylated or not) may affect any of these processes. Gaining a clearer understanding of these processes in future studies may elucidate the biological processes that drive hydroxylated sphingomyelins to be top predictors of antidepressant treatment outcomes among the metabolites that were assayed.

Laboratory investigation of predictive metabolites (e.g., hydroxylated sphingomyelins) could begin with the repeatedly validated metabolomics-informed-genomics approach [28]. The success of this approach in discovering novel MDD or antidepressant response biology is exemplified by our identification and functional investigation of the *TSPAN5*, *ERICH3*, *DEFB1*, and *AHR* genes. SNPs in or near these genes associated with concentrations of serotonin or kynurenine, which were in turn associated with citalopram/escitalopram treatment outcomes or baseline depression in a PGRN-AMPS cohort [24, 25, 27]. The metabolomics-informed-genomics studies, which identified those genes utilized a liquid chromatography electrochemical coulometric array (LC-ECA) metabolomics platform [27]. The LC-ECA platform quantified 31 known plasma metabolites in PGRN-AMPS patients, primarily in the tryptophan, tyrosine, purine, and tocopherol pathways [27]. In contrast, the 153 metabolites included in this work were quantified utilizing the p180 platform, which detects up to 40 acylcarnitines, 42 amino acids and biogenic amines, 90 glycerophospholipids, 15 sphingolipids, and sum of hexoses. The expanse of metabolites assayed by the p180 platform metabolites have previously improved predictability of change in QIDS self-reported score [13]. By combining SNPs of established predictability arising from LC-ECA detected metabolites with the expanse of metabolites available from the p180 platform, this work examines for the first time the joint predictability of these independently predictive biomarkers.

Compared with the electrochemical coulometric array platform, which quantified 31 plasma metabolites in previous work [27], the p180 platform detected 153 metabolites after quality control in this work. Therefore, we required a novel strategy to identify metabolites for follow-up mechanistic investigation. Linear associations between the 31 LC-ECA assayed metabolites and outcomes were previously successful in identifying serotonin and kynurenine for mechanistic investigation, linear associations between the 153 p180-assayed metabolites and outcomes yield many more candidate metabolites than can be immediately pursued in laboratory-based studies. The analytical strategy outlined here provides an alternative approach suited for expansive assays to identify metabolites for functional studies.

This study also highlights the clinical importance of developing better means to predict antidepressant treatment outcomes (i.e., incorporating biological markers for improving predictability and achieving cross-trial replications) in depressed patients [8, 55]. Monotherapy with antidepressants is the recommended first-line treatment approach. However, poor or incomplete responses to monotherapy occur frequently, even at optimized doses [56]. Antidepressant combinations are important strategies to consider in such circumstances [57]. Not surprisingly, antidepressant combinations, including the combinations studied in this work, are frequently encountered in clinical practice [58–62]. The field has begun to investigate clinically accessible parameters (such as BMI and markers of systemic inflammation, such as C-reactive protein), which have shown promise as predictive biomarkers for antidepressive effects with serotonergic and non-serotonergic antidepressants [63–65]. However, there are still no widely implemented evidence-based methods for predicting treatment outcomes with antidepressant combinations, either at the start of treatment or based on early (e.g., 4 weeks) response to therapy. Future studies that focus on integrating biological and symptom-based factors to predict clinical outcomes with other commonly used antidepressant combinations are needed. Supporting the validity of using a multi-omics machine learning process for predicting clinical outcomes is the ability of the models to generalize in diverse populations.

The top predictors varied by algorithm and feature set, as can be expected given the different mathematical underpinnings of penalized regression and boosting algorithms. In cross-validation, elastic net regression was demonstrated to be the optimal penalized regression approach for both the metabolomics and multi-omics data sets. Elastic net regression simultaneously performs continuous shrinkage and variable selection and is particularly useful for selecting predictive groups of correlated variables [66]. Elastic net regression determined sphingomyelins, phosphatidylcholines, and carnitines to be top metabolite predictors (Fig. 2A). In contrast to elastic net regression, boosting algorithms are tree-based approaches. They first split the decision trees on the feature that best classifies the training samples, then they iteratively reduce erroneous classifications by exploring the joint predictive capabilities of other features [67]. Given that we know that early change in MDD severity associates with eventual treatment outcomes [68], it is likely that XGBoost begins classification based on early MDD severity change, thereby making this a top predictor in its models (Fig. 2B), before considering the contributions of other predictors in the final adjudication of whether a patient responds or not. Neither penalized regression nor boosting provides a definitively superior set of top predictors—jointly assessing the top predictors of both approaches demonstrates that hydroxylated sphingomyelins are consistently amongst top predictors. Therefore, investigating hydroxylated sphingomyelins in future work mechanistic work may lead to improved insights into the biology of antidepressant response.

In addition, non-White race was a top predictor in the multi-omics penalized regression model, but not in the metabolomics penalized regression model (Fig. 2A). This may have arisen from correlations between SNPs and race, i.e., differences in SNP minor allele frequencies among populations (Supplementary Table 8). These SNPs were initially discovered in the context of MDD in the PGRN-AMPS cohort, a primarily white population, which may explain their interaction with non-white race in the penalized regression models. Future work should aim to establish the predictability of these SNPs, along with any SNPs arising from mechanistic investigation of hydroxylated sphingomyelins, across populations.

Performance metrics were lower in the training data set compared to the testing data set (Table 2 and Supplementary Tables 3 and 4). This may have arisen from (a) higher inter-patient variability in measures in the training data set and/or (b) smaller sample sizes in testing data sets that may comprise subjects with lesser inter-patient variability in disease severity or drug response. Although the current results are encouraging in that multi-omics models developed with monotherapy patients established predictability of outcomes to combination antidepressant therapy, future work with additional biomarkers (e.g., neuroimaging, additional genomic SNPs, and proteomics) may achieve comparable performances in both larger and smaller sized studies.

This work has limitations. Lipids assayed by current mass spectrometry technology may actually reflect sum signals of all isomeric/isobaric compounds having the same parent and daughter ions, and that phenomenon may occur with the hydroxylated sphingomyelins [69]. Therefore, future approaches should validate the identified variables with other assays. Although eventual response may be predicted as early as 2 weeks post-treatment initiation [70], this was not feasible in this work due to lack of data at week 2. Minor allele frequencies for the included SNPs vary by population (Supplementary Table 8), so larger samples with individuals of diverse ancestry will be needed to validate prediction performance across populations. Although fasting status may not significantly affect laboratory variability for most metabolites [71], we cannot exclude the possibility of bias introduced by lack of fasting samples [72, 73]. This work’s analysis includes only the clinical/sociodemographic features common to both cohorts, so relevant features including clinical comorbidities, socioeconomic status, and number of depressive episodes were not examined. In addition, high missingness (>35%) in BMI and serotonin concentration data, stemming from the absence of collection and quantifications below the lower limit of detection or lowest calibration standard, respectively, precluded their incorporation in these analyses. Both studies excluded patients with a history of psychotic symptoms, so these findings may not generalize to patients with psychotic depression. Finally, we did not have blood drug levels for all patients, but this limitation is less concerning given that drug levels did not associate with clinical outcomes in previous studies [7, 10].

In summary, this is the first study that demonstrates improved predictability of antidepressant treatment outcomes in depressed adults receiving combination antidepressant therapy by augmenting clinical measures with multiple biological measures. The integration of multiple biological markers (e.g., SNPs and metabolites) suggested the prognostic importance of hydroxylated sphingomyelins in the context of monoamine neurotransmission. The current workflow ranks biomarkers according to their contributions to improved predictability of antidepressant response and provides a foundation for future laboratory-based studies, which may identify novel molecular mechanisms of MDD pathophysiology, drug response, or both.

## Supplementary information

Supplementary Materials

## Data Availability

All raw and analyzed data and related materials, including programming code, are available upon request to Mayo Clinic Ventures or University of Texas Southwestern. PGRN-AMPS data have been deposited on dbGaP, study accession phs000670.v1.p.1
