# Supplementary material for: Multi-omics driven predictions of response to acute phase combination antidepressant therapy: a machine learning approach with cross-trial replication
Source: Transl Psychiatry. 2021 Oct 7;11:513. doi: 10.1038/s41398-021-01632-z (PMC8497535; doi:10.1038/s41398-021-01632-z)
Supplement: Supplementary file 1 — Supplementary Materials [file 41398_2021_1632_MOESM1_ESM.docx]

**Supplementary Materials**

**TITLE: Multi-Omics Driven Predictions of Response to Acute Phase Combination Antidepressant Therapy: A Machine Learning Approach with Cross-Trial Replication**

**PREVIOUS PRESENTATION:** None

**Running title**: Multi-Omics Predictions of Antidepressant Outcomes

**AUTHORS:** Jeremiah Joyce*, M.D., M.S.^1^, Caroline Grant*, B.A.^2^, Duan Liu, Ph.D.^2^, Siamak MahmoudianDehkordi, Ph.D.^3^, Rima Kaddurah-Daouk, Ph.D.^3^, Michelle Skime^1^, Joanna Biernacka, Ph.D.^4^, Mark A. Frye, M.D.^1^, Taryn Mayes M.S.^5^, Thomas Carmody, Ph.D.^6^, Paul E. Croarkin D.O., M.S.^1^, Liewei Wang, M.D., Ph.D.^2^, Richard Weinshilboum, M.D.^2^, William V. Bobo, M.D., M.P.H.^7^, Madhukar H. Trivedi M.D.^5^, Arjun P. Athreya M.S., Ph.D.^2^

**AUTHOR AFFILIATIONS**

1. Department of Psychiatry and Psychology, Mayo Clinic, Rochester, MN, USA.

2. Department of Molecular Pharmacology and Experimental Therapeutics, Mayo Clinic, Rochester, MN, USA.

3. Department of Psychiatry and Behavioral Sciences, Department of Medicine, Duke Institute for Brain Sciences, Duke University, Durham, NC, USA.

4. Department of Quantitative Health Sciences, Mayo Clinic, Rochester, MN, USA.

5. Peter O’Donnell Jr. Brain Institute and the Department of Psychiatry at the University of Texas Southwestern Medical Center, Dallas, TX, USA.

6. Department Population and Data Sciences at the University of Texas Southwestern Medical Center in Dallas

7. Department of Psychiatry and Psychology, Mayo Clinic, Jacksonville, FL, USA.

*These authors contributed equally to this work.

**Corresponding author:**

Arjun P. Athreya, M.S., PhD.,

Dept. of Molecular Pharmacology and Exp. Therapeutics,

Mayo Clinic,

200 First St. SW,

Rochester, MN – 55902

Tel: +1-507-422-6073

Email: [athreya.arjun@mayo.edu](mailto:athreya.arjun@mayo.edu)

**FIGURES**

**Figure Legends**

**Supplementary Figure 1.** Sample inclusion by study.

**Supplementary Figure 2.** Multi-omics integration network excluding kynurenine, implemented in xMWAS. This analysis was conducted to determine whether community membership was maintained in the absence of the known strong correlations between kynurenine and the *AHR* and *DEFB1* SNPs. The results were not perturbed based on inclusion/exclusion of kynurenine. Metabolite names corresponding to these numbers can be found in Supplementary Table 3.

**Tables**

**Table Legends**

**Supplementary Table 1.** Clinical and sociodemographic features of samples from PGRN-AMPS and CO-MED studies. *Significantly different (p < 0.05) between studies or between splits according to Mann-Whitney U or chi-square tests. † Ethnicity characterization in PGRN-AMPS is based off data from 205 out of the 264 patients, based upon availability of data.

**Supplementary Table 2.** Model features. Week 4 percent delta is calculated as (QIDS-C week 4 total - QIDS-C baseline total) / (QIDS-C baseline total). Week 4 delta is calculated as (QIDS-C week 4 total) - (QIDS-C baseline total). Hexoses and lipids are analyzed by flow-injection analysis-tandem mass spectrometry (FIA-MS/MS). The FIA-MS/MS signals represent the sum signals of all isomeric/isobaric compounds having the same parent and daughter ions. Biocrates provides a full list of isomeric/isobaric compounds (<https://biocrates.com/wp-content/uploads/2020/02/Biocrates_Q500_isomers_isobars.pdf>).

**Supplementary Table 3:** Model metrics from models augmenting the six metabolomic-informed- pharmacogenomic SNPs with the additional rs10245483 SNP relating to P-glycoprotein expression.

**Supplementary Table 4:** Model metrics using three-fold and ten-fold cross validation strategies, with three repeats. Models were trained and tested using all available SSRI monotherapy-treated patients (citalopram/escitalopram/escitalopram+placebo).

**Supplementary Table 5:** Model hyperparameters. For penalized regression performed via glmnet in R, the ‘mixture’ hyperparameter of 0.8 or 0.6 indicates tuning selected elastic net regression. A mixture of 0 represents ridge regression, a mixture of 1 represents lasso regression, and values in between 0 and 1 represent elastic net regression.

**Supplementary Table 6.** Metabolite labels for integrative network analysis.

**Supplementary Table 7.** Metabolite-SNP correlations for integrative network analysis. Weight represents the Pearson correlation.

**Supplementary Table 8.** Population frequencies for included SNPs according to the gnomAD v2.1.1 browser (https://gnomad.broadinstitute.org).

**Supplementary Figure 1: Sample inclusion by study**

Yes

Yes

Yes

PGRN-AMPS: 529

CO-MED: 665

QIDS-C data at weeks 0, 4, 8?

Baseline metabolomics?

< 20% individual data missing?

Genotyping?

**Metabolomics Set**

PGRN-AMPS: 264

CO-MED: 111

**Multi-Omics Set**

PGRN-AMPS: 245

CO-MED: 103

No

No

No

No

PGRN-AMPS: 0

CO-MED: 233

PGRN-AMPS: 265

CO-MED: 320

PGRN-AMPS: 0

CO-MED: 1

PGRN-AMPS: 19

CO-MED: 8

Training: 277

Testing:71

Training: 298

Testing:77

**Supplementary Figure 2: xMWAS Analysis Excluding Kynurenine**

**
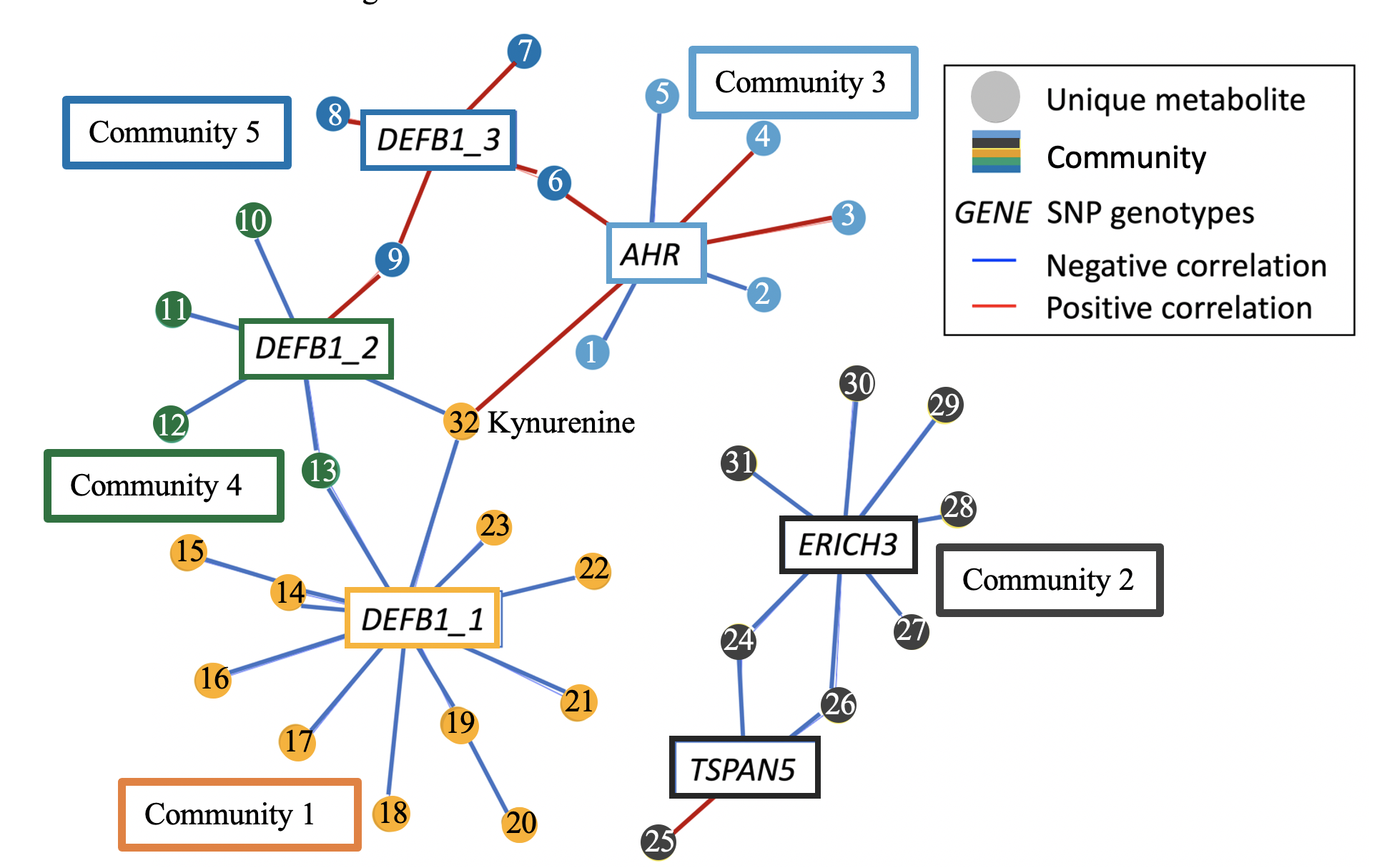
**

| **Supplementary Table 1: Sociodemographic and Clinical Features Common to PGRN and COMED** | | |
| --- | --- | --- |
| Sociodemographic Features | PGRN-AMPS  N = 264 | CO-MED  N = 111 |
| Sex [% Female] | 65.5% | 71.2% |
| Age [mean (SD)] | 39.6 (13.1)* | 45.0 (11.9)* |
| Years of education [mean (SD)] | 14.8 (2.3)* | 13.9 (2.8)* |
| Race [% White] | 97.3%* | 73.2%* |
| Race [% Black or African American] | 1.1%* | 18.8%* |
| Race [% Other] | 1.6%* | 8.0%* |
| Ethnicity [% Hispanic] | 1.5%†* | 19.8%* |
| **Clinical Features** |  |  |
| Depression onset before age 18 [%] | 42.8% | 42.3% |
| Prior Suicide Attempts [N (%)] | 44 (16.7%)* | 9 (8.1%)* |
| QIDS-C at baseline [mean (SD)] | 15 (3.3) | 15.7 (3.6) |
| QIDS-C Response at Week 4 | 47.3% | 41.4% |
| QIDS-C Remission at Week 4 | 27.2% | 23.4% |
| QIDS-C Response at Week 8 | 68.9% | 61.3% |
| QIDS-C Remission at Week 8 | 48.5% | 40.5% |

| **Supplementary Table 2: Predictors Included in Model Sets 1 and 2** | | | |
| --- | --- | --- | --- |
| **Predictor**  **Category** | **Collection**  **Timepoint** | **Predictors** | **Model Model Set 1 Set 2** |
| Clinical Clinical Clinical Clinical Clinical  Clinical | Baseline, Week 4 % Change Baseline, Week 4 Delta Baseline, Week 4 Delta Baseline, Week 4 Delta Baseline, Week 4 Delta  Baseline, Week 4 Delta | QIDS-C Total 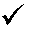 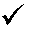 QIDS-C item - Sad Mood 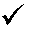 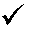 QIDS-C item – Psychomotor Slowing 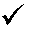 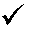 QIDS-C item - Self-Outlook 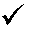 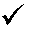 QIDS-C item - Involvement 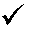 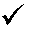  QIDS-C item - Fatigue 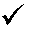 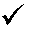 | |
| Clinical | Baseline, Week 4 Delta | QIDS-C item - Psychomotor Slowing 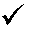 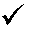 | |
| Clinical | Baseline | Prior Suicide Attempt 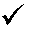 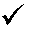 | |
| Clinical | Baseline | Depression onset before age 18 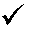 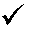 | |
| Sociodemographic | Baseline | Age 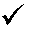 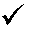 | |
| Sociodemographic | Baseline | Sex 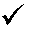 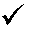 | |
| Sociodemographic | Baseline | Ethnicity 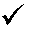 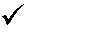 | |
| Sociodemographic | Baseline | Years of Education 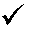 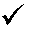 | |
| Metabolomic | Baseline | Alanine 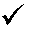 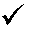 | |
| Metabolomic | Baseline | Arginine 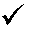 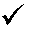 | |
| Metabolomic | Baseline | Asparagine 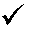 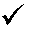 | |
| Metabolomic | Baseline | Aspartate 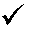 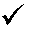 | |
| Metabolomic | Baseline | Citrulline 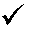 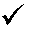 | |
| Metabolomic | Baseline | Glutamine 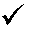 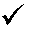 | |
| Metabolomic | Baseline | Glutamate 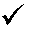 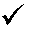 | |
| Metabolomic | Baseline | Glycine 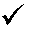 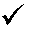 | |
| Metabolomic | Baseline | Histidine 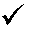 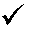 | |
| Metabolomic | Baseline | Isoleucine 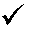 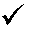 | |
| Metabolomic | Baseline | Lysine 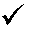 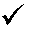 | |
| Metabolomic | Baseline | Methionine 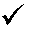 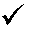 | |
| Metabolomic | Baseline | Ornithine 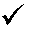 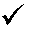 | |
| Metabolomic | Baseline | Phenylalanine 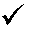 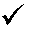 | |
| Metabolomic | Baseline | Proline 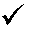 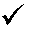 | |
| Metabolomic | Baseline | Serine 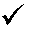 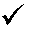 | |
| Metabolomic | Baseline | Threonine 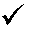 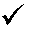 | |
| Metabolomic | Baseline | Tryptophan 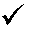 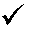 | |
| Metabolomic | Baseline | Tyrosine 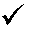 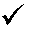 | |
| Metabolomic | Baseline | Valine 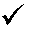 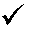 | |
| Metabolomic | Baseline | Asymmetric Dimethylarginine 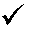 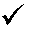 | |
| Metabolomic | Baseline | Kynurenine 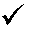 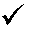 | |
| Metabolomic | Baseline | Methioninesulfoxide 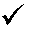 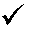 | |

| Metabolomic | Baseline | Symmetric Dimethylarginine |
| --- | --- | --- |
| Metabolomic | Baseline | Spermidine |
| Metabolomic | Baseline | Spermine |
| Metabolomic | Baseline | Hydroxyproline |
| Metabolomic | Baseline | Taurine |
| Metabolomic | Baseline | lysoPhosphatidylcholine acyl C16:0 |
| Metabolomic | Baseline | lysoPhosphatidylcholine acyl C16:1 |
| Metabolomic | Baseline | lysoPhosphatidylcholine acyl C17:0 |
| Metabolomic | Baseline | lysoPhosphatidylcholine acyl C18:0 |
| Metabolomic | Baseline | lysoPhosphatidylcholine acyl C18:1 |
| Metabolomic | Baseline | lysoPhosphatidylcholine acyl C18:2 |
| Metabolomic | Baseline | lysoPhosphatidylcholine acyl C20:3 |
| Metabolomic | Baseline | lysoPhosphatidylcholine acyl C20:4 |
| Metabolomic | Baseline | lysoPhosphatidylcholine acyl C24:0 |
| Metabolomic | Baseline | lysoPhosphatidylcholine acyl C26:0 |
| Metabolomic | Baseline | lysoPhosphatidylcholine acyl C26:1 |
| Metabolomic | Baseline | lysoPhosphatidylcholine acyl C28:0 |
| Metabolomic | Baseline | lysoPhosphatidylcholine acyl C28:1 |
| Metabolomic | Baseline | Phosphatidylcholine diacyl C24:0 |
| Metabolomic | Baseline | Phosphatidylcholine diacyl C28:1 |
| Metabolomic | Baseline | Phosphatidylcholine diacyl C30:0 |
| Metabolomic | Baseline | Phosphatidylcholine diacyl C32:0 |
| Metabolomic | Baseline | Phosphatidylcholine diacyl C32:1 |
| Metabolomic | Baseline | Phosphatidylcholine diacyl C32:3 |
| Metabolomic | Baseline | Phosphatidylcholine diacyl C34:1 |
| Metabolomic | Baseline | Phosphatidylcholine diacyl C34:2 |
| Metabolomic | Baseline | Phosphatidylcholine diacyl C34:3 |
| Metabolomic | Baseline | Phosphatidylcholine diacyl C34:4 |
| Metabolomic | Baseline | Phosphatidylcholine diacyl C36:1 |
| Metabolomic | Baseline | Phosphatidylcholine diacyl C36:2 |
| Metabolomic | Baseline | Phosphatidylcholine diacyl C36:3 |
| Metabolomic | Baseline | Phosphatidylcholine diacyl C36:4 |
| Metabolomic | Baseline | Phosphatidylcholine diacyl C36:5 |
| Metabolomic | Baseline | Phosphatidylcholine diacyl C36:6 |
| Metabolomic | Baseline | Phosphatidylcholine diacyl C38:0 |
| Metabolomic | Baseline | Phosphatidylcholine diacyl C38:3 |
| Metabolomic | Baseline | Phosphatidylcholine diacyl C38:4 |
| Metabolomic | Baseline | Phosphatidylcholine diacyl C38:5 |
| Metabolomic | Baseline | Phosphatidylcholine diacyl C38:6 |
| Metabolomic | Baseline | Phosphatidylcholine diacyl C40:2 |


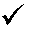

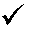

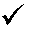

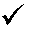

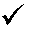

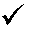

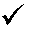

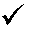

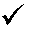

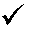

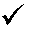

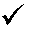

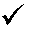

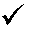

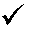

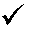

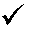

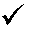

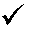

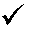

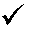

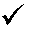

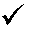

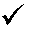

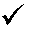

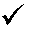

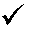


| Metabolomic | Baseline | Phosphatidylcholine diacyl C40:3 |
| --- | --- | --- |
| Metabolomic | Baseline | Phosphatidylcholine diacyl C40:4 |
| Metabolomic | Baseline | Phosphatidylcholine diacyl C40:5 |
| Metabolomic | Baseline | Phosphatidylcholine diacyl C40:6 |
| Metabolomic | Baseline | Phosphatidylcholine diacyl C42:0 |
| Metabolomic | Baseline | Phosphatidylcholine diacyl C42:1 |
| Metabolomic | Baseline | Phosphatidylcholine diacyl C42:2 |
| Metabolomic | Baseline | Phosphatidylcholine diacyl C42:4 |
| Metabolomic | Baseline | Phosphatidylcholine diacyl C42:5 |
| Metabolomic | Baseline | Phosphatidylcholine diacyl C42:6 |
| Metabolomic | Baseline | Phosphatidylcholine acyl alkyl C30:0 |
| Metabolomic | Baseline | Phosphatidylcholine acyl alkyl C30:2 |
| Metabolomic | Baseline | Phosphatidylcholine acyl alkyl C32:1 |
| Metabolomic | Baseline | Phosphatidylcholine acyl alkyl C32:2 |
| Metabolomic | Baseline | Phosphatidylcholine acyl alkyl C34:0 |
| Metabolomic | Baseline | Phosphatidylcholine acyl alkyl C34:1 |
| Metabolomic | Baseline | Phosphatidylcholine acyl alkyl C34:2 |
| Metabolomic | Baseline | Phosphatidylcholine acyl alkyl C34:3 |
| Metabolomic | Baseline | Phosphatidylcholine acyl alkyl C36:0 |
| Metabolomic | Baseline | Phosphatidylcholine acyl alkyl C36:1 |
| Metabolomic | Baseline | Phosphatidylcholine acyl alkyl C36:2 |
| Metabolomic | Baseline | Phosphatidylcholine acyl alkyl C36:3 |
| Metabolomic | Baseline | Phosphatidylcholine acyl alkyl C36:4 |
| Metabolomic | Baseline | Phosphatidylcholine acyl alkyl C36:5 |
| Metabolomic | Baseline | Phosphatidylcholine acyl alkyl C38:0 |
| Metabolomic | Baseline | Phosphatidylcholine acyl alkyl C38:2 |
| Metabolomic | Baseline | Phosphatidylcholine acyl alkyl C38:3 |
| Metabolomic | Baseline | Phosphatidylcholine acyl alkyl C38:4 |
| Metabolomic | Baseline | Phosphatidylcholine acyl alkyl C38:5 |
| Metabolomic | Baseline | Phosphatidylcholine acyl alkyl C38:6 |
| Metabolomic | Baseline | Phosphatidylcholine acyl alkyl C40:1 |
| Metabolomic | Baseline | Phosphatidylcholine acyl alkyl C40:2 |
| Metabolomic | Baseline | Phosphatidylcholine acyl alkyl C40:3 |
| Metabolomic | Baseline | Phosphatidylcholine acyl alkyl C40:4 |
| Metabolomic | Baseline | Phosphatidylcholine acyl alkyl C40:5 |
| Metabolomic | Baseline | Phosphatidylcholine acyl alkyl C40:6 |
| Metabolomic | Baseline | Phosphatidylcholine acyl alkyl C42:1 |
| Metabolomic | Baseline | Phosphatidylcholine acyl alkyl C42:2 |
| Metabolomic | Baseline | Phosphatidylcholine acyl alkyl C42:3 |
| Metabolomic | Baseline | Phosphatidylcholine acyl alkyl C42:4 |

| Metabolomic | Baseline | Phosphatidylcholine acyl alkyl C42:5 |
| --- | --- | --- |
| Metabolomic | Baseline | Phosphatidylcholine acyl alkyl C44:3 |
| Metabolomic | Baseline | Phosphatidylcholine acyl alkyl C44:4 |
| Metabolomic | Baseline | Phosphatidylcholine acyl alkyl C44:5 |
| Metabolomic | Baseline | Phosphatidylcholine acyl alkyl C44:6 |
| Metabolomic | Baseline | Sphingomyelin (OH) C14:1 |
| Metabolomic | Baseline | Sphingomyelin (OH) C16:1 |
| Metabolomic | Baseline | Sphingomyelin (OH) C22:1 |
| Metabolomic | Baseline | Sphingomyelin (OH) C22:2 |
| Metabolomic | Baseline | Sphingomyelin (OH) C24:1 |
| Metabolomic | Baseline | Sphingomyelin C16:0 |
| Metabolomic | Baseline | Sphingomyelin C16:1 |
| Metabolomic | Baseline | Sphingomyelin C18:0 |
| Metabolomic | Baseline | Sphingomyelin C18:1 |
| Metabolomic | Baseline | Sphingomyelin C20:2 |
| Metabolomic | Baseline | Sphingomyelin C24:0 |
| Metabolomic | Baseline | Sphingomyelin C24:1 |
| Metabolomic | Baseline | Sphingomyelin C26:0 |
| Metabolomic | Baseline | Sphingomyelin C26:1 |
| Metabolomic | Baseline | Carnitine (C0) |
| Metabolomic | Baseline | Decanoylcarnitine (C10) |
| Metabolomic | Baseline | Decadienylcarnitine (C10:2) |
| Metabolomic | Baseline | Tetradecenoylcarnitine (C14:1) |
| Metabolomic | Baseline | Hydroxytetradecenoylcarnitine (C14:1-OH) |
| Metabolomic | Baseline | Tetradecadienylcarnitine (C14:2) |
| Metabolomic | Baseline | Hydroxytetradecadienylcarnitine (C14:2-OH) |
| Metabolomic | Baseline | Hexadecanoylcarnitine (C16) |
| Metabolomic | Baseline | Hexadecenoylcarnitine (C16:1) |
| Metabolomic | Baseline | Hydroxyhexadecenoylcarnitine (C16:1-OH) |
| Metabolomic | Baseline | Hydroxyhexadecanoylcarnitine (C16-OH) |
| Metabolomic | Baseline | Octadecanoylcarnitine (C18) |
| Metabolomic | Baseline | Octadecenoylcarnitine (C18:1) |
| Metabolomic | Baseline | Hydroxyoctadecenoylcarnitine (C18:1-OH) |
| Metabolomic | Baseline | Octadecadienylcarnitine (C18:2) |
| Metabolomic | Baseline | Acetylcarnitine (C2) |
| Metabolomic | Baseline | Propionylcarnitine (C3) |
| Metabolomic | Baseline | Propenonylcarnitine (C3:1) |
| Metabolomic | Baseline | Hydroxybutyrylcarnitine (C3-DC (C4-OH)) |
| Metabolomic | Baseline | Hydroxypropionylcarnitine (C3:oh) |
| Metabolomic | Baseline | Butyrylcarnitine (C4) |

| Metabolomic Metabolomic Metabolomic Metabolomic Metabolomic Metabolomic Metabolomic Metabolomic Metabolomic  Metabolomic | Baseline Baseline Baseline Baseline Baseline Baseline Baseline Baseline Baseline  Baseline | Butenylcarnitine (C4:1) Valerylcarnitine (C5) Tiglylcarnitine (C5:1) Glutaconylcarnitine (C5:1-DC) Glutarylcarnitine (C5-DC (C6-OH))  Hydroxyvalerylcarnitine (C5-OH (C3-DC-M)) Hexenoylcarnitine (C6:1)  Hexanoylcarnitine (C6 (C4:1-DC)) Pimelylcarnitine (C7-DC)  Nonaylcarnitine (C9) |
| --- | --- | --- |
| Genomic | Baseline | TSPAN5 (rs10516436) |
| Genomic | Baseline | ERICH3 (rs696692) |
| Genomic | Baseline | AHR (rs17137566) |
| Genomic | Baseline | DEFB1_1 (rs5743467) |
| Genomic | Baseline | DEFB1_2 (rs2741130) |
| Genomic | Baseline | DEFB1_3 (rs2702877) |

| **Supplementary Table 3: Prediction Model Metrics incorporating the P-Glycoprotein rs10245483 SNP** | | | | |
| --- | --- | --- | --- | --- |
| **2A: Models trained with PGRN-AMPS escitalopram, PGRN-AMPS citalopram, and CO-MED escitalopram+placebo patients.** | | | | |
|  | **Model Set 1 (Metabolomic)** | | **Model Set 2 (Multi-Omics)** | |
|  | **XGBoost** | **Penalized Regression** | **XGBoost** | **Penalized Regression** |
| **Testing-Set Metrics** | | | | |
| **AUC** | 0.76 | 0.85 | 0.83 | 0.86 |
| **Accuracy** | 0.73 | 0.77 | 0.76 | 0.77 |
| **NIR** | 0.62 | 0.62 | 0.63 | 0.63 |
| **p-value** | 0.053 | 0.0045 | 0.017 | 0.0067 |
| **Sensitivity** | 0.75 | 0.69 | 0.69 | 0.71 |
| **Specificity** | 0.69 | 0.90 | 0.88 | 0.88 |
| **Training-Set Metrics (in cross validation)** | | | | |
| **AUC** | 0.69 | 0.69 | 0.68 | 0.72 |
| **Accuracy** | 0.64 | 0.66 | 0.67 | 0.65 |
| **NIR** | 0.68 | 0.68 | 0.69 | 0.69 |
| **p-value** | 0.57 | 0.79 | 0.89 | 0.57 |
| **2B: Models trained with PGRN-AMPS escitalopram and PGRN-AMPS citalopram patients.** | | | | |
| **Testing-Set Metrics** | | | | |
| **AUC** | 0.75 | 0.84 | 0.75 | 0.86 |
| **Accuracy** | 0.75 | 0.75 | 0.73 | 0.77 |
| **p-value** | 0.026 | 0.026 | 0.085 | 0.0067 |
| **Sensitivity** | 0.65 | 0.73 | 0.82 | 0.71 |
| **Specificity** | 0.93 | 0.79 | 0.57 | 0.88 |
| **Training-Set Metrics (in cross validation)** | | | | |
| **AUC** | 0.68 | 0.68 | 0.72 | 0.72 |
| **Accuracy** | 0.64 | 0.65 | 0.67 | 0.68 |
| **NIR** | 0.69 | 0.69 | 0.70 | 0.70 |
| **p-value** | 0.084 | 0.14 | 0.31 | 0.55 |

| **Supplementary Table 4: Extended Prediction Model Metrics** | | | | |
| --- | --- | --- | --- | --- |
| **4A: Metrics using 3-Fold Cross-Validation with 3 Repeats** | | | | |
|  | **Model Set 1 (Metabolomic)** | | **Model Set 2 (Multi-Omics)** | |
|  | **XGBoost** | **Penalized Regression** | **XGBoost** | **Penalized Regression** |
| **Testing-Set Metrics** | | | | |
| **AUC** | 0.68 | 0.85 | 0.71 | 0.86 |
| **Accuracy** | 0.64 | 0.77 | 0.66 | 0.77 |
| **NIR** | 0.62 | 0.62 | 0.63 | 0.63 |
| **p-value** | 0.81 | 0.0045 | 0.62 | 0.0067 |
| **Sensitivity** | 0.63 | 0.69 | 0.8 | 0.71 |
| **Specificity** | 0.66 | 0.9 | 0.42 | 0.88 |
| **Training-Set Metrics (in cross validation)** | | | | |
| **AUC** | 0.66 | 0.69 | 0.72 | 0.69 |
| **Accuracy** | 0.64 | 0.65 | 0.7 | 0.63 |
| **NIR** | 0.68 | 0.68 | 0.69 | 0.69 |
| **4B: Metrics Using 10-Fold Cross-Validation with 3 Repeats** | | | | |
|  | **Model Set 1 (Metabolomic)** | | **Model Set 2 (Multi-Omics)** | |
|  | **XGBoost** | **Penalized Regression** | **XGBoost** | **Penalized Regression** |
| **Testing-Set Metrics** | | | | |
| **AUC** | 0.78 | 0.85 | 0.81 | 0.86 |
| **Accuracy** | 0.73 | 0.79 | 0.75 | 0.77 |
| **NIR** | 0.62 | 0.62 | 0.63 | 0.63 |
| **p-value** | 0.054 | 0.0006 | 0.04 | 0.0067 |
| **Sensitivity** | 0.81 | 0.75 | 0.58 | 0.71 |
| **Specificity** | 0.59 | 0.86 | 0.84 | 0.88 |
| **Training-Set Metrics (in cross validation)** | | | | |
| **AUC** | 0.7 | 0.69 | 0.71 | 0.72 |
| **Accuracy** | 0.66 | 0.66 | 0.68 | 0.66 |
| **NIR** | 0.68 | 0.68 | 0.69 | 0.69 |

| **Supplementary Table 5: Prediction Model Hyperparameters** | | | | |
| --- | --- | --- | --- | --- |
| **2A: Models trained with PGRN-AMPS escitalopram, PGRN-AMPS citalopram, and CO-MED escitalopram+placebo patients.** | | | | |
|  | **Model Set 1 (Metabolomic)** | | **Model Set 2 (Multi-Omics)** | |
|  | **XGBoost** | **Penalized Regression** | **XGBoost** | **Penalized Regression** |
| **Hyperparameters** | trees = 822 | penalty = 0.14 | trees = 807 | penalty = 0.14 |
|  | min_n = 14 | mixture = 0.80 | min_n = 10 | mixture = 0.8 |
|  | trees_depth = 3 |  | trees_depth = 1 |  |
|  | learn rate = 1.1e-5 |  | learn rate = 1.8e-5 |  |
|  | loss reduction=5.4e-5 |  | loss reduction = 1.60e-3 |  |
|  | sample size = 0.55 |  | sample size = 0.43 |  |
| **2B: Models trained with PGRN-AMPS escitalopram and PGRN-AMPS citalopram patients.** | | | | |
|  | **Model Set 1 (Metabolomic)** | | **Model Set 2 (Multi-Omics)** | |
|  | **XGBoost** | **Penalized Regression** | **XGBoost** | **Penalized Regression** |
| **Hyperparameters** | trees = 579 | penalty = 0.14 | trees = 1133 | penalty = 0.14 |
|  | min_n = 23 | mixture = 0.6 | min_n = 5 | mixture = 1 |
|  | trees_depth = 3 |  | trees_depth = 3 |  |
|  | learn rate = 4.20e-5 |  | learn rate = 4.36e-5 |  |
|  | loss reduction = 0.0066 |  | loss reduction = 1.06e-6 |  |
|  | sample size = 0.90 |  | sample size = 0.62 |  |

| **Supplementary Table 6: xMWAS Metabolite Labels** | | |
| --- | --- | --- |
| **Label** | **Community** | **Metabolite** |
| 1 | 3 | Tetradecenoylcarnitine |
| 2 | 3 | Tetradecadienylcarnitine |
| 3 | 3 | Phenylalanine |
| 4 | 3 | Lysine |
| 5 | 3 | Phosphatidylcholine acyl alkyl C44:6 |
| 6 | 5 | Histidine |
| 7 | 5 | Methionine |
| 8 | 5 | Threonine |
| 9 | 5 | Asparagine |
| 10 | 4 | Phosphatidylcholine diacyl C30:0 |
| 11 | 4 | Phosphatidylcholine diacyl C32:1 |
| 12 | 4 | Glutarylcarnitine |
| 13 | 4 | Phosphatidylcholine diacyl C34:3 |
| 14 | 1 | Phosphatidylcholine diacyl C36:1 |
| 15 | 1 | Carnitine |
| 16 | 1 | Asymmetric dimethylarginine |
| 17 | 1 | Butyrylcarnitine |
| 18 | 1 | Citrulline |
| 19 | 1 | Phosphatidylcholine diacyl C36:2 |
| 20 | 1 | Phosphatidylcholine diacyl C36:5 |
| 21 | 1 | Phosphatidylcholine diacyl C34:1 |
| 22 | 1 | Phosphatidylcholine diacyl C36:3 |
| 23 | 1 | Phosphatidylcholine diacyl C34:2 |
| 24 | 2 | Taurine |
| 25 | 2 | Tyrosine |
| 26 | 2 | Aspartate |
| 27 | 2 | Sphingomyelin C24:1 |
| 28 | 2 | Sphingomyelin C16:1 |
| 29 | 2 | Sphingomyelin-(OH)-C16:1 |
| 30 | 2 | Sphingomyelin C26:0 |
| 31 | 2 | Sphingomyelin C26:1 |
| 32 | 1 | Kynurenine |

| **Supplementary Table 7: Correlations (Weight) of Integrative Network Analysis Connections** | | |
| --- | --- | --- |
| **From** | **To** | **Weight** |
| Asymmetric dimethylarginine | rs5743467 (DEFB1_1) | -0.112 |
| Asparagine | rs2741130 (DEFB1_2) | 0.115 |
| Asparagine | rs2702877 (DEFB1_3) | 0.125 |
| Aspartate | rs696692 (ERICH3) | -0.139 |
| Aspartate | rs10516436 (TSPAN5) | -0.131 |
| Carnitine | rs5743467 (DEFB1_1) | -0.111 |
| Tetradecenoylcarnitine | rs17137566 (AHR) | -0.151 |
| Tetradecadienylcarnitine | rs17137566 (AHR) | -0.126 |
| Butyrylcarnitine | rs5743467 (DEFB1_1) | -0.132 |
| Glutarylcarnitine | rs2741130 (DEFB1_2) | -0.116 |
| Citrulline | rs5743467 (DEFB1_1) | -0.151 |
| Histidine | rs17137566 (AHR) | 0.113 |
| Histidine | rs2702877 (DEFB1_3) | 0.113 |
| Kynurenine | rs5743467 (DEFB1_1) | -0.266 |
| Kynurenine | rs2741130 (DEFB1_2) | -0.144 |
| Kynurenine | rs17137566 (AHR) | 0.134 |
| Lysine | rs17137566 (AHR) | 0.125 |
| Methionine | rs2702877 (DEFB1_3) | 0.112 |
| Phosphatidylcholine Diacyl C30:0 | rs2741130 (DEFB1_2) | -0.139 |
| Phosphatidylcholine Diacyl C32:1 | rs2741130 (DEFB1_2) | -0.131 |
| Phosphatidylcholine Diacyl C34:1 | rs5743467 (DEFB1_1) | -0.129 |
| Phosphatidylcholine Diacyl C34:2 | rs5743467 (DEFB1_1) | -0.114 |
| Phosphatidylcholine Diacyl C34:3 | rs5743467 (DEFB1_1) | -0.133 |
| Phosphatidylcholine Diacyl C34:3 | rs2741130 (DEFB1_2) | -0.117 |
| Phosphatidylcholine Diacyl C36:1 | rs5743467 (DEFB1_1) | -0.131 |
| Phosphatidylcholine Diacyl C36:2 | rs5743467 (DEFB1_1) | -0.121 |
| Phosphatidylcholine Diacyl C36:3 | rs5743467 (DEFB1_1) | -0.12 |
| Phosphatidylcholine Diacyl C36:5 | rs5743467 (DEFB1_1) | -0.116 |
| Phosphatidylcholine Acyl Alkyl C44:6 | rs17137566 (AHR) | -0.112 |
| Phenylalanine | rs17137566 (AHR) | 0.128 |
| Sphingomyelin C16:1 | rs696692 (ERICH3) | -0.117 |
| Sphingomyelin C24:1 | rs696692 (ERICH3) | -0.154 |
| Sphingomyelin C26:0 | rs696692 (ERICH3) | -0.116 |
| Sphingomyelin C26:1 | rs696692 (ERICH3) | -0.117 |
| Sphingomyelin-(OH)-C16:1 | rs696692 (ERICH3) | -0.123 |
| Taurine | rs10516436 (TSPAN5) | -0.135 |
| Taurine | rs696692 (ERICH3) | -0.121 |
| Threonine | rs2702877 (DEFB1_3) | 0.133 |
| Tyrosine | rs10516436 (TSPAN5) | 0.137 |

| **Supplementary Table 8: Population frequencies for included SNPs** | | | | | | |
| --- | --- | --- | --- | --- | --- | --- |
|  | TSPAN5  (rs10516436) | ERICH3  (rs696692) | AHR  (rs17137566) | DEFB1_1  (rs5743467) | DEFB1_2  (rs2741130) | DEFB1_3  (rs2702877) |
| African/African American | 0.21 | 0.88 | 0.17 | 0.067 | 0.35 | 0.67 |
| Latino | 0.16 | 0.66 | 0.18 | 0.17 | 0.40 | 0.68 |
| Other | 0.099 | 0.61 | 0.18 | 0.23 | 0.48 | 0.74 |
| European (non-Finnish) | 0.076 | 0.62 | 0.16 | 0.25 | 0.44 | 0.73 |
| Ashkenazi Jewish | 0.068 | 0.63 | 0.18 | 0.26 | 0.36 | 0.86 |
| East Asian | 0.062 | 0.43 | 0.38 | 0.0025 | 0.42 | 0.74 |
| European (Finnish) | 0.05 | 0.57 | 0.17 | 0.24 | 0.57 | 0.74 |
